# Eptesipox virus-associated lesions in naturally infected big brown bats

**DOI:** 10.1177/03009858241231556

**Published:** 2024-02-17

**Authors:** Ursula G. Perdrizet, Janet E. Hill, Champika Fernando, LaRhonda Sobchishin, Vikram Misra, Trent K. Bollinger

**Affiliations:** 1 University of Saskatchewan, Saskatoon, SK, Canada

**Keywords:** big brown bat, chiroptera, eptesipox virus, joint, oral ulcer, poxviridae, ultrastructure, whole genome sequencing

## Abstract

Bats have many unique qualities amongst mammals; one of particular importance is their reported tolerance to viruses without developing disease. Here, the authors present evidence to the contrary by describing and demonstrating viral nucleic acids within lesions from eptesipox virus (EfPV) infection in big brown bats. One hundred and thirty bats submitted for necropsy from Saskatchewan, Canada, between 2017 and 2021 were screened for EfPV by polymerase chain reaction (PCR); 2 had amplifiable poxvirus DNA. The lesions associated with infection were oral and pharyngeal ulcerations and joint swelling in 2/2 and 1/2 cases, respectively. These changes were nonspecific for poxvirus infection, although intracytoplasmic viral inclusion bodies within the epithelium, as observed in 2/2 bats, are diagnostic when present. Viral nucleic acids, detected by in situ hybridization (ISH), were observed in the epithelium adjacent to ulcerative lesions from both cases and within the joint proliferation of 1 case. A new isolate of EfPV was obtained from 1 case and its identity was confirmed with electron microscopy and whole genome sequencing. Juxtanuclear replication factories were observed in most cells; however, rare intranuclear virus particles were also observed. The significance of the presence of virus particles within the nucleus is uncertain. Whole genome assembly indicated that the nucleotide sequence of the genome of this EfPV isolate was 99.7% identical to a previous isolate from big brown bats in Washington, USA between 2009 and 2011. This work demonstrates that bats are not resistant to the development of disease with viral infections and raises questions about the dogma of poxvirus intracytoplasmic replication.

Bats have been thought of as having a special relationship with viruses where they are more resistant to infection-associated disease.^43^ Much of the current research relies on next-generation sequencing to identify viruses of bats, but there are few studies examining the pathological effects of infection in the host^5,11,19,26,29,41,50^ and only a few reports from experimental inoculations.^6,9,23,28,30,42^

Poxviruses are double-stranded DNA viruses that replicate in the cytoplasm and encode many proteins that modulate the host response to infection. Poxviruses appear to have acquired some of these modulating genes from their hosts, by horizontal gene transfer.^22^ Ulceration and proliferation of the epithelium are clinical manifestations common to many poxviruses.^35^ Infections are sometimes more extensive and affect multiple organs, such as the wet form of avipox infections in birds or myxoma virus infections in domestic rabbits.^35^ The difference in lesions is due to the variability in cellular permissivity to poxviruses and the variability in the degree of viral replication in infected cells.^36^

Poxviruses have been found in bats from around the world and are most often discovered by sequencing data but are occasionally associated with lesions. Evidence in Yinpterochiroptera (megabats) of poxvirus infection include partial poxviral sequences from swabs in Ghanian *Eidolon helvum*,^1^ whole genome sequencing for pteropox virus associated with multiple crusting lesions on the wings of *Pteropus scapulatus* in Australia,^40^ and a virus isolated from nodular wing lesions in *Rousettus aegyptiacus* from Israel named Israeli *Rousettus aegyptiacus* poxvirus.^8^ Similar evidence is also available for Yangochiroptera (microbats), such as the identification of Hypsugopoxvirus by sequencing from *Hypsugo savii* in Italy,^33^ nodular skin lesions with viral inclusion bodies and virions visible on electron micrograph from *Miniopterus schreibersii bassanii* in Australia,^37^ and isolation and sequencing of eptesipox virus (EfPV) from the big brown bat (*Eptesicus fuscus*) in the United States with fibrinosuppurative, necrotizing tenosynovitis and osteoarthritis.^12,54^

The objectives of this research were to examine wild bats submitted for necropsy to the Western/Northern Regional Centre of the Canadian Wildlife Health Cooperative for evidence of lesions associated with viral infection, to isolate and identify virus(es), and to determine whether there is an association of observed pathological changes with viral replication.

## Materials and Methods

### Case Description and Diagnostics

The first diagnosed case of EfPV from which virus was isolated was an adult male big brown bat submitted 8 July 2020. This bat had been relinquished to a wildlife rehabilitation unit for inability to fly and had multiple swollen joints that did not improve with 10 days of supportive care. The second case, also an adult male big brown bat, had been submitted 13 May 2019, after being cared for by wildlife rehabilitators for 74 days with a chronic nonhealing ulcer over the nose. Intracytoplasmic inclusions were noted in the pharynx with histopathological examination and a poxvirus infection was suspected but no further diagnostics were pursued.

Joints from the index case were submitted for aerobic and anaerobic culture and a *Mycoplasma* species polymerase chain reaction (PCR). The PCR is commercially available through Prairie Diagnostic Services Inc. Briefly, the DNA was extracted using MagMax Core Nucleic Acid Purification Kit (Thermofisher cat. A32700). A semi-nested PCR was performed as previously described^56^ using 5 U/μl Taq DNA polymerase recombinant (ThermoScientific cat. EP0406) and 10 mM each dNTP mix (ThermoScientific cat. R0192). The product was purified with Qiagen PCR purification kit (Qiagen cat. 28104) and sequenced by the National Research Council, University of Saskatchewan.

### Screening Cases for EfPV

Following the submission of the index case of EfPV from Saskatchewan, tissues from previous and ongoing submissions for gammaherpesvirus surveillance within the province were also screened for poxvirus infection using conventional PCR. There were 294 big brown bats submitted for necropsy during this period, and of these we received 130 samples. Each sample was from an individual case and comprised pooled liver, lung, and/or spleen, as available. Of the remaining cases, samples were not received for a variety of reasons, including necropsies were performed but tissues were not collected, tissue samples were not suitable due to advanced decomposition or scavenging, or no necropsy was performed. DNA was extracted from the liver, lung, and spleen using the DNeasy blood and tissue kit (Qiagen, cat. 69504). For the PCR, HotStarTaq (Qiagen, cat. 203205) was used following the manufacturer’s protocol, which involved denaturation at 95°C × 15 minutes, 40 cycles of denaturation at 94°C × 30 seconds, annealing at 62°C × 39 seconds, and extension at 72°C × 1 minutes with a final extension at 72°C × 10 minutes. The following primers that targeted the type A inclusion protein and generated a 765 bp product were used: forward: 5′-GACGAACACGATGCATCACG-3′ and reverse: 5′-TAGTGGAGGTAGCGGTGGA-3′.

### Histopathology

Tissues were fixed in 10% neutral-buffered formalin, paraffin-embedded, sectioned at 5 µm, mounted on glass slides, and stained with hematoxylin and eosin using standard protocols (Supplemental Table S1). Sections containing bone, which included sections of joints and oral mucosa, were decalcified after fixation by immersion in 20% formic acid for 24 hours, prior to embedding. Slides were reviewed using an Olympus BX41 microscope and images were captured using an Olympus BX41, Infinity 5 camera, and Infinity Analyze version 7.0.3.1111 software.

### In Situ Hybridization

Unstained, 5 μm tissue sections from formalin-fixed paraffin-embedded tissue blocks were mounted on Superfrost Plus slides (Fisher Scientific cat. 12-550-15). They were probed using the RNAScope 2.5 HD Assay Brown kit (ACDbio cat. 322300) following the manufacturer’s protocol with pretreatment in the target retrieval buffer for 30 minutes at 95°C–100°C and 15 minutes for protease plus treatment at 40°C for all tissues. Probes were designed to target viral nucleic acids encoding the p39 putative membrane-associated core protein and the p4b precursor protein, genes *gp99* and *gp100*, respectively (ACDbio cat. 1070961-C1). Two viral gene targets were used because of the lack of diversity in the EfPV genome. Serial sections of slides were probed with a positive technical control targeting the *peptidyl-prolyl isomerase B variant X1* gene of big brown bats (Ef-PPIB-C1 cat. 1073191-C1) and a negative control probe targeting the *dihydrodipicolinate reductase* gene from bacteria (negative control probe DapB cat. 310043).

### Virus Isolation

From the index case, pooled liver, lung, and spleen or a swollen joint frozen at –20°C were homogenized in 1.5 ml of Dulbecco’s modified medium (Gibco, cat. 12430112) at 30 Hz for 4 minutes in a Retsch Mixer Mill with a 5.5 mm stainless steel grinding bead (MP Biomedicals, 116540431) and 0.1 g of silica beads (Fisher, cat. 360991112). The samples were centrifuged at 15,700 × *g* × 15 minutes. One milliliter of each of the 2 supernatants were added to their own respective 75 cm^2^ flasks (Sarstedt, cat. 83.3911.002) of passage 12 *Eptesicus fuscus* kidney cell line 3b (EfK3b) cells in 4 ml of Dulbecco’s modified medium and incubated for 1 hour at 37°C. After incubation, Dulbecco’s modified medium containing 10% fetal bovine serum, penicillin, streptomycin, and amphotericin B (antibiotic-antimycotic, Gibco, cat. 5240062) was added to the flask. Seven days postinfection, 95% cytopathic effect was observed in one of the flasks, and flasks were frozen at –80°C. This isolate was named eptesipox virus/Saskatoon/01/2020 (EfPV/SK).

### Electron Microscopy and Virus Purification

Cells from the spindle proliferation in the metacarpophalangeal joint of the index case were lifted from an unstained serial section of the slide and prepared as previously described.^2^ Ultrathin 90 nm sections were cut and stained with uranyl acetate.

Virus was purified from four 175 cm^2^ flasks of EfK3b cells inoculated at a multiplicity of infection of 0.001 with EfPV. When 90% cytopathic effect was observed the supernatant was frozen and thawed 3 times followed by serial centrifugation at 4°C: 1,500 × *g* × 5 minutes (Sorvall Legend RT, Thermo Scientific), 10,000 × *g* × 15 minutes (Sorvall RC6 Plus, Thermo Scientific, Waltham), and 80,000 × *g* × 1 hour (Sorvall Wx Ultra, Thermo Scientific). The pellet was resuspended in 40 μl of phosphate-buffered saline with 20 μl of 2% glutaraldehyde in 0.1 M sodium cacodylate and stored at 4°C until imaging. Electron microscopy was performed as previously described.^51^ Virus was purified in a similar manner for genome sequencing by ultracentrifugation with 6 ml of 30% w/v sucrose cushion and resuspended in 50 μl of 10 mM Tris pH 8.5.

For cellular electron microscopy, one 175 cm^2^ flask of EfK3b cells was inoculated with 10 µl of purified virus diluted in 5 ml of Dulbecco’s modified medium and incubated for 1 hour at 37°C, then complete media was added. After 18, 24, or 36 hours, media and trypsinized cells were centrifuged at 325 × *g* × 10 minutes (Sorvall legend, Thermo Scientific). The pellet was resuspended in 36 ml of phosphate-buffered saline and centrifugation was repeated. Cells were fixed with 10 ml of 2% glutaraldehyde in 0.1 M sodium cacodylate and incubated at 4°C for 4 hours. They were pelleted with the previous centrifugation step and resuspended in 1 ml of 0.1M sodium cacodylate. Images were captured with the Hitachi HT7700 transmission electron microscope and measurements were taken with Image-Pro Premier version 9.3.3.

### Genome Sequencing

DNA was extracted from purified virus using the DNeasy blood and tissue kit (Qiagen, cat. 69504). The sequencing library was constructed using Nextera XT Library Preparation kit (Illumina, cat. FC-131-1024) according to the manufacturer’s protocol. An 8 picomolar library was sequenced using a Miseq platform in 2 × 250 cycles using a Miseq V2 500 cycle Nano kit (Illumina, cat. MS-103-1003). Reads were trimmed using Trimmomatic^3^ sliding window 4:30 and minlen 36, resulting in 2501x coverage of the retained reads for EfPV/SK. Quality filtered reads were assembled into contigs using SPAdes 3.12.0^39^ and mapped to the reference genome eptesipox virus strain Washington (EfPV/WA) NC_035460 using Geneious Prime 2022.0.1. Gaps between contiguous sequences were resolved using HotStarTaq PCR, using products purified with MinElute PCR purification kit (Qiagen, cat. 28004) or gel extraction and purification with the QIAquick gel extraction kit (Qiagen, cat. 28706), followed by Sanger sequencing (Macrogen). The TOPO TA Cloning (Invitrogen, cat. K45000-40) was used to clone the PCR product from the largest gap into the topo vector and submitted for sequencing by Plasmidsaurus (Eugene, OR). The 56,461 contiguous sequences generated from the short reads were checked for similarity against the viruses’ nucleotide database (National Center for Biotechnology Information) using megaBLAST.^57^ Any sequences with significant similarity were then queried against the entire nucleotide database using the same method (National Center for Biotechnology Information).

### Phylogenetic Tree

The concatenated amino acid sequences from the following viral proteins were aligned using MUSCLE in Geneious Prime 2022.0.1: RPO132, RPO147, VETF-L, RAP94, mRNA capping enzyme large subunit, P4a precursor, P4b, DNA topoisomerase I, VLTF-2, NPH-II, Holliday junction resolvase, DNA packaging ATPase, and DNA primase. The poxviruses that were used included: EfPV/SK OM638613, eptesipox virus Washington strain NC_035460, hypsugopox 251170-23/2017 MK860688, pteropox NC_030656, cowpox Germany 1980 EP4 HQ420895, vaccinia WR AY243312, variola NC_001611, camelpox NC_003391, taterapox NC_008291, raccoonpox NC_027213, orf NC_005336, Yaba monkey tumor NC_005179, rabbit fibroma NC_001266, deerpox W-848-83 NC_006966, swinepox NC_003389, sheeppox 17077-99 NC_004002, cotia SPAn232 NC_016924, canarypox NC_005309, and salmon gillpox NC_027707. Choristoneura biennis entomopoxvirus ‘L’ was used as the outgroup, NC_021248.1. The maximum likelihood method and Jones-Taylor-Thornton model was used to construct a phylogenetic tree on the concatenated amino acid sequence alignments using PhyML 3.3.20180621 with 1,000 bootstrap replicates.^18^

### Supplementary Materials

Please see the online version for access to supplementary files.

### Availability of Supporting Research Data

The sequencing data analyzed in this study is available at GenBank, repository number OM638613.

## Results

### Diagnostics

In July 2020, a big brown bat was submitted for multiple joint swellings from which EfPV was isolated, hereafter referred to as the index case. Bacterial culture of this case yielded few *Serratia liquefaciens* and a *Staphylococcus* species was isolated. The PCR for *Mycoplasma* was positive. The closest similarity to the amplicon in a megaBLAST search to the nucleotide database (National Center of Biotechnology Information) was *Mycoplasma procyoni* with 96.76% identity.

### Screening Cases for EfPV

A retrospective search of the Canadian Wildlife Health Cooperative database for similar reports of viral infection identified one other case of suspected poxviral infection from 2019. DNA extracted from pooled liver, lung, and spleen samples available from 130 big brown bats was screened for poxvirus using PCR. These cases were submitted between 2017 and 2021, and the 130 bats screened were a subsample of 294 big brown bats submitted during this time. These 130 bats were selected based on tissue availability. Poxvirus DNA was detected by PCR in 2/130 cases, including the index case. Only 3 samples were tested from 2021. No previous record of this virus was found within the Canadian Wildlife Health Cooperative database.

### Histopathology

**Figure 1.**
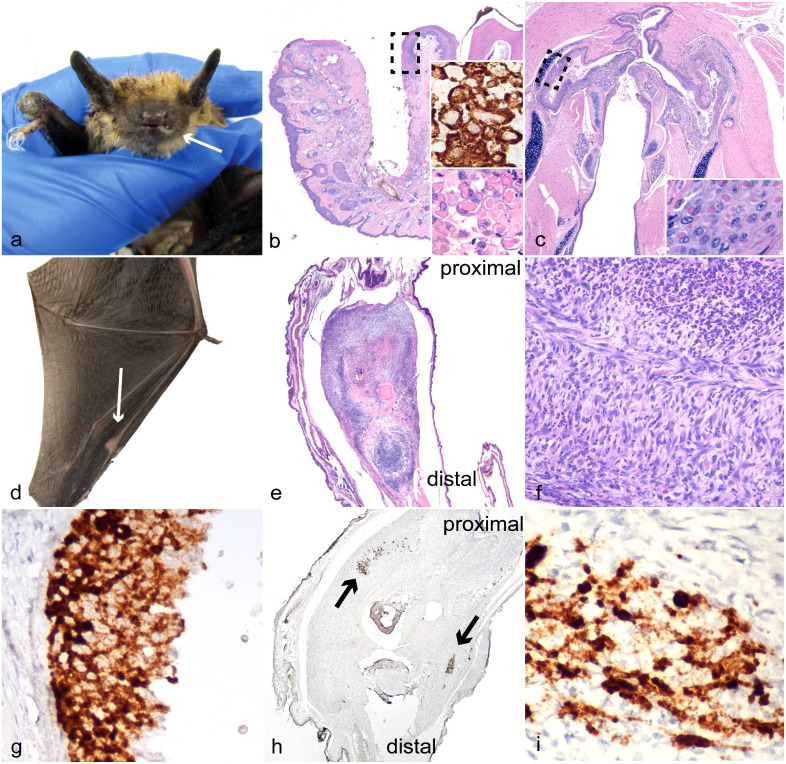
Big brown bat eptesipox virus-associated lesions. (a, b, d–f, h, i) Index case from which virus was isolated. (a) Left lower lip. Gross image of swelling and ulceration (arrow) and swelling of right carpus. (b) Lower left lip and mandible. Ulcer of lip and gingiva, the dashed box represents the location of higher magnification inset. Hematoxylin and eosin (HE). Inset (bottom): higher magnification of keratinocytes with intracytoplasmic inclusion bodies. HE. Inset (top): eptesipox virus (EfPV) in situ hybridization (ISH) probe binding is predominantly restricted to the cytoplasm. (c) Pharynx. Extensive ulceration in the second big brown bat identified with EfPV infection, the dashed box represents the location of higher magnification inset. HE. Inset: higher magnification of intracytoplasmic pink viral inclusion bodies in the epithelium. HE (d) Metacarpophalangeal joint of the third digit of the left wing with joint swelling (arrow). (e) Left third metacarpophalangeal joint that is expanded by spindle cell proliferation. HE. (f) Left third metacarpophalangeal joint. Higher magnification showing large numbers of neutrophils amongst the spindle cells. HE. (g) Pharynx. Serial section of the ulcerated pharyngeal epithelium in (c) with probe binding restricted primarily to the cytoplasm. EfPV ISH. (h) Left third metacarpophalangeal joint. A serial section of the joint in (e) with multifocal probe binding to EfPV nucleic acids (arrows). EfPV ISH. (i) Left third metacarpophalangeal joint. Higher magnification demonstrating more intense binding in the nucleus than the cytoplasm of spindle cells. EfPV ISH.

To characterize the lesions associated with infection, the hematoxylin and eosin-stained slides from both PCR positive cases were reviewed in addition to 14 other cases that had previously tested negative for EfPV by PCR. The 14 controls were selected based on the presence of similar lesions, types of tissue on slides, recentness of submission, and preservation of tissue (Supplemental Table S1). Of the 15 bats where oral and/or nasal mucosa was examined, ulcers were present in the 2 positive cases (Fig. 1a) and 4 of 13 controls. One of the controls did not have mucosa available for review. The oral and pharyngeal ulcers in the 2 cases in which EfPV was detected contained large pink intracytoplasmic inclusion bodies typical of poxvirus infections (Fig. 1b, c). Oral ulcerations in the control cases did not have this distinguishing feature. Ulcerations and proliferations of the epidermis of the wing were present in 1 of the EfPV cases, but no inclusion bodies were observed. Wing ulcerations were found in 3 of 14 controls, one of the EfPV cases did not have wing available for examination. In the case of poxviral infection with multiple swollen joints, all were affected by a moderate to severe neutrophilic infiltrate within the surrounding tissues and joint space, sometimes mixed with fibrin. In addition to this inflammation, several joints had synovium thickened by spindle to stellate cells (fibroblast-like synoviocytes) (Fig. 1d–f). In the previous report of EfPV infection, necrosis was a predominant histologic finding but in our case, it was only present in the elbow joint and the surrounding muscles and tendons.^12^ No inclusion bodies were observed in the joint lesions. Joints were only available for examination in 4 controls; 3 of these had lesions with neutrophilic infiltrates as well as thickening of the synovium, the other was normal. One of the control cases with joint lesions also had ulceration of the nasal mucosa with large colonies of bacteria in the joint and nasal lesions. The stomach was evaluated in 14 bats, and lesions were observed in 1 of 2 EfPV infected bats and 4 of 12 controls, which consisted of multifocal erosions or, in 1 case, ulcerations. An additional lesion present in 4/13 controls, but not in EfPV cases, was focal to multifocal liver necrosis; a lesion that has been reported in cases of poxvirus infection.^35^ For one of the control cases, the liver was not available for examination. Gross lesions of joint swelling and epidermal or mucosal ulcerations were not specific to poxvirus infections. Intracytoplasmic inclusion bodies were the most consistent diagnostic feature, but these were not present in all lesions.

### In Situ Hybridization

To determine the association of viral nucleic acids with lesions, the same 16 cases reviewed for pathological changes were subjected to ISH. Probe binding for the viral genes encoding the structural proteins membrane-associated core protein and p4b precursor was only present in the mucosal and joint lesions from the PCR positive cases (Fig. 1b, g–i). There was no probe binding in the controls with or without similar lesions. Probe binding within the metacarpophalangeal joint was intense and obscured the nucleus and cellular features, preventing the identification of the cell type. The ISH for EfPV was specific for EfPV nucleic acids and demonstrated the virus was associated with synovial proliferations and mucosal ulcerations. Images of the positive technical control for the metacarpophalangeal joint of the index case, and the EfPV probe for a selection of controls, can be found in Supplemental Figure S1.

### Electron Microscopy and Virus Isolation

**Figure 2.**
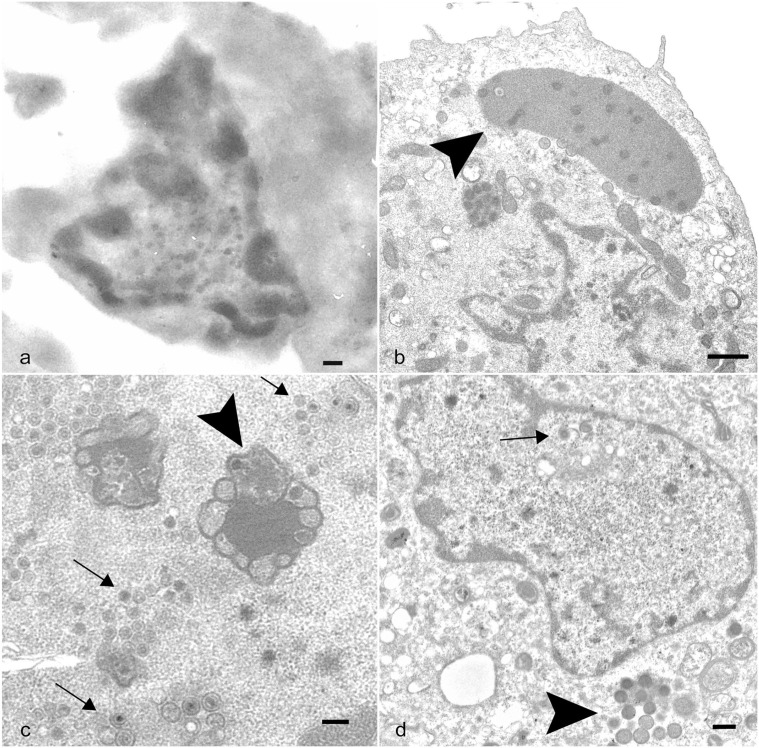
Big brown bat eptesipox virus transmission electron micrographs of joint proliferation and cell culture. (a) Left third metacarpophalangeal joint. Transmission electron micrograph (TEM) of a spindle cell from an unstained serial section of the joint with a mottled nucleus containing round to oblong structures with an electron-lucent outer layer and an electron-dense core. Scale bar: 200 nm. (b–d) Eptesicus fuscus kidney cell line 3b cells infected with eptesipox virus, TEM. (b) Cell with large juxtanuclear replication factories in the cytoplasm (arrowhead) characteristic of poxviral infections. Scale bar: 1 µm. (c) Cell with intranuclear structures consistent with viral factories (arrowhead) and virions (arrows). Scale bar: 200 nm. (d) Cell with intranuclear virus core and capsids (arrow) and intracytoplasmic immature virions (arrowhead). Scale bar: 400 nm.

Transmission electron microscopy was used to identify any viral or virus-like particles in the joint tissue from the index case and confirm the type of virus isolated from the same bat. Spindle cells from an unstained serial section of the metacarpophalangeal joint that were lifted from the areas with EfPV ISH probe binding contained numerous round to oblong structures within the nucleus that were suggestive of viral particles (Fig. 2a). Infected cultured cells displayed juxtanuclear replication factories in the cytoplasm characteristic of poxvirus infection (Fig. 2b). The negatively stained purified virus had the characteristic size and shape of poxvirus, having a brick shape and measuring 222 nm long by 195 nm wide. At 36 hours post infection intranuclear viral replication was observed in one cell (Fig. 2c). Repeating the transmission electron microscopy at different time points demonstrated no intranuclear virus at 18 hours post infection but at 24 hours post infection, 2 in approximately 15 cells had both intranuclear immature virions, viral capsid with a core, and evidence of intracytoplasmic replication (Fig. 2d). The diameters of the capsids in the nucleus were 118 ± 36 nm, whereas the intracytoplasmic immature virions were 248 ± 16 nm.

### Genome Sequencing

A *de novo* assembly of short read data was performed to determine whether this was a new species or matched the reference sequence for EfPV (NC_035460). The isolate named eptesipox virus/Saskatoon/01/2020 shared 99.7% nucleotide identity to the EfPV Washington strain NC_035460 indicating the isolate was EfPV.^54^ The sequence was deposited in GenBank accession number OM638613. Between the 2 isolates, there were 5 deletions, 3 insertions, 89 transitions, 17 transversions, 5 substitutions, and 16 insertions or deletions in tandem repeats. These variations were less frequently present in the center of the genome between 60,000 and 120,000 bp. Of the 56,461 contiguous sequences generated with SPAdes, 93 had significant similarities to viruses when queried against the viruses nucleotide database; this excludes contiguous sequences with similarity to EfPV. When these sequences were queried against the full nucleotide database, the majority were more similar to host sequences or a closely related bat species than viral sequences. Nine of these sequences did not have similarity to any bat genomes and the closest query was from various mammals and bacteria at a greater degree of similarity than viral sequences. A 457 bp sequence did yield a significant match to a virus, bovine parvovirus 3.

### Phylogenetic Analysis

**Figure 3.**
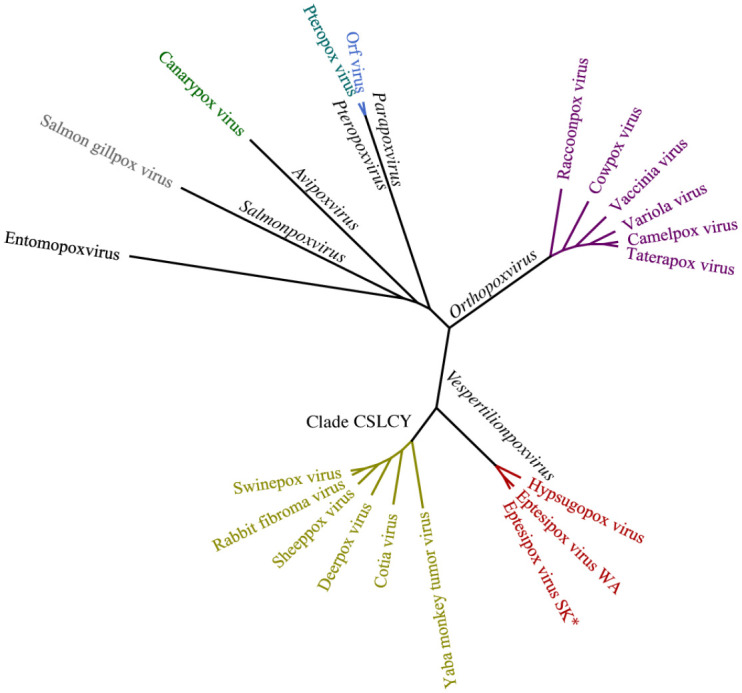
Maximum likelihood phylogenetic tree analysis of poxviruses using concatenated amino acid alignments of 13 genes rooted on an entomopoxvirus. All other viruses belong to the *Chordopoxvirinae* subfamily and genera of the branches are in black. The new isolate eptesipox virus/Saskatoon/01/2020 is indicated by an asterisk (*). CSCLY, Capripoxvirus, Suipoxvirus, Leporipoxvirus, Cervidpoxvirus, and Yatapoxvirus genera; WA, Washington; SK, Saskatoon.

A phylogenetic tree was constructed because of genetic differences in the new isolate of EfPV and to include another poxvirus from the same host genus *Eptesicus*, which led us to confirm the relationship of EfPV to other bat poxviruses (Fig. 3).^12,40,54^ Proteins were chosen based on previous phylogenies of other members of the Poxviridae family.^21,40,54^ Of the 13 EfPV protein sequences examined, only 2 differed in their amino acid sequence from the reference sequence NC_035460: the mRNA capping enzyme large subunit (1/844 amino acids) and P4a (1/908 amino acids). Although the difference in the mRNA capping enzyme may not represent a true difference because a nucleic acid could not be resolved.

## Discussion

Our study illustrates that bats can develop significant disease associated with EfPV infections and, although not severe enough to directly result in death, ultimately these lesions could be fatal due to impaired flight, impaired feeding, and/or secondary infections. Some of the lesions displayed in these cases are typical for poxviruses, such as proliferation and ulceration of the epithelium, but there are also atypical lesions in the joints and periarticular tissues. Infection with certain species of poxviruses in specific hosts can result in a systemic infection with variable necrotizing or proliferative lesions in multiple internal organs, but this was not observed in these 2 cases.^27,35^ Joint lesions, as seen in one of these cases, and as described in the initial EfPV report, have not been described in other poxviral infections, except for variola virus infections. Osteomyelitis variolosa is the term for this uncommon presentation of smallpox (variola) infection.^53^ Detailed histopathological descriptions of osteomyelitis variolosa are lacking, but some have reported epiphyseal involvement, primarily affecting children.^52,53^ The joint lesions in bats with EfPV were primarily synovial and periarticular; detailed description of the parts of the bone affected in the original report are missing.^12^ There appears to be systemic involvement with EfPV infection (joint lesions), and there is potentially a large spectrum of lesions and clinical manifestations that are unrecognized.

Several of the affected joints showed limited proliferative change and were negative by ISH, which may indicate the pathogenesis is not identical amongst the affected joints or could reflect differing stages of infection. Probe binding to poxvirus has a multifocal patchy distribution in the oral cavity and one joint; therefore, when no binding was observed in other joints, this could be an artifact of sectioning where the virus is missed in the section. We also do not know the contribution of *Mycoplasma* to the joint lesions although to date no pathology has been associated with a similar species of *Mycoplasma* and it may be a commensal organism.^55^

Despite a previous in vitro demonstration of multiple cellular mechanisms in bats that dampen the innate and inflammatory response to DNA, we observed a marked inflammatory response in affected joints.^17^ Neutrophils display protective effects against viruses but can also contribute to disease, primarily through tissue destruction by the release of granule contents.^15^ Experimental poxviral infections have induced strong neutrophil responses.^25^ Poxviruses can inhibit leukocyte migration through chemokine binding proteins. These have been shown to either selectively inhibit monocyte chemokines, or the chemokines of both monocytes and neutrophils, interfering with migration of these leukocytes.^31,48^ Based on the large numbers of neutrophils in the joints and lack of other leukocytes, the function of the EfPV chemokine binding protein is proposed to be selective inhibition of monocyte migration.

Our unexpected observation of intranuclear viral replication is not unusual for the nucleocytoplasmic large DNA viruses to which poxviruses belong.^10,44^ Of this group, the only other viruses to replicate exclusively in the cytoplasm are mimiviruses.^38,47^ The lack of nuclear involvement in poxvirus replication has made for a conundrum in how poxviruses acquire host genes through horizontal gene transfer.^14^ The majority of horizontal gene transfer events between viruses and eukaryotes have been identified in the nucleocytoplasmic large DNA viruses, including poxviridae, supporting some type of nuclear involvement in their replication.^24^ An alternative method of horizontal gene transfer is the inclusion of host genes via reverse transcription, and this method is supported by the fact that poxviral genes lack introns.^4^ However, these introns could be lost through different mechanisms.^34^ Intranuclear viral replication requires confirmation and may be an artifact of the cell line or cell culture. Electron micrographs of penguin poxvirus infections have also demonstrated intranuclear immature viral particles in mammalian cells,^49^ although these findings could be artifacts secondary to the breakdown of the nuclear envelope. However, light microscopy has provided evidence for nuclear involvement in poxvirus infections by identifying intranuclear inclusions.^13,16,45,46^ Although intranuclear inclusions are suggestive of viral infection they do not always reflect active viral replication as seen in the electron micrograph of the skin nevus (freckle) associated with molluscum contagiosum virus.^46^ Since the virus particles in the nucleus were smaller than what we observed in cytoplasmic replication, we cannot rule out the possibilities of co-isolation of a virus that replicates in the nucleus like a herpesvirus, or contamination of the viral culture. However, if there was a second virus, we would have expected to detect this in the sequencing data, which we did not. The bat from which EfPV was isolated tested negative via PCR for Eptesicus fuscus gammaherpesvirus.

Whole genome sequencing identified our viral isolate as a previously described EfPV.^12,54^ There were differences between the sequences and some of these changes altered the amino acids of predicted proteins that could result in functional changes between these isolates. The International Committee on Taxonomy of Viruses recently introduced the genus *Vespertilionpoxvirus* to the Poxviridae family, which includes EfPV as the sole species.^32^ Hypsugopox virus is closely related to EfPV and currently remains unclassified. It likely belongs in the same genus as similarities in the aligned amino acid sequences range from 76.7% to 95.1% for individual proteins and 88.7% for the concatenated sequences (Fig. 3). The hosts of these viruses, *Eptesicus fuscus* and *Hypsugo savii*, belong to the same *Vespertilioninae* subfamily despite living on different continents.^20^

The limitations of this study are the small sample size and a design that does not allow us to determine a causal relationship between viral infection and the lesions observed. The lesions observed in infected bats are not typical of poxvirus infections in other species but share similarities to clinical forms of variola infection in humans. Although joint involvement seems a prominent feature of infection this may be because these bats were found debilitated by members of the public, had easily observed lesions, and were cared for by rehabilitators, which likely increased their survival. The spectrum of disease associated with EfPV infection may be broader than what is currently described because of their reclusive and nocturnal nature.^7^ Further research is required to better understand the role of EfPV in the lesions observed and in bat health.

## Supplemental Material

sj-pdf-1-vet-10.1177_03009858241231556 – Supplemental material for Eptesipox virus-associated lesions in naturally infected big brown bats Supplemental material, sj-pdf-1-vet-10.1177_03009858241231556 for Eptesipox virus-associated lesions in naturally infected big brown bats by Ursula G. Perdrizet, Janet E. Hill, Champika Fernando, LaRhonda Sobchishin, Vikram Misra and Trent K. Bollinger in Veterinary Pathology
