# Supplementary material for: Eptesipox virus-associated lesions in naturally infected big brown bats
Source: Vet Pathol. 2024 Feb 17;61(4):541–9. doi: 10.1177/03009858241231556 (PMC11264557; doi:10.1177/03009858241231556)
Supplement: sj-pdf-1-vet-10.1177_03009858241231556 – Supplemental material for Eptesipox virus-associated lesions in naturally infected big brown bats [file sj-pdf-1-vet-10.1177_03009858241231556.pdf]

Supplemental Materials

Eptesipox virus-associated lesions in naturally infected big brown bats

Ursula G Perdrizet, Janet E Hill, Champika Fernando, LaRhonda Sobchishin, Vikram Misra, Trent K Bollinger

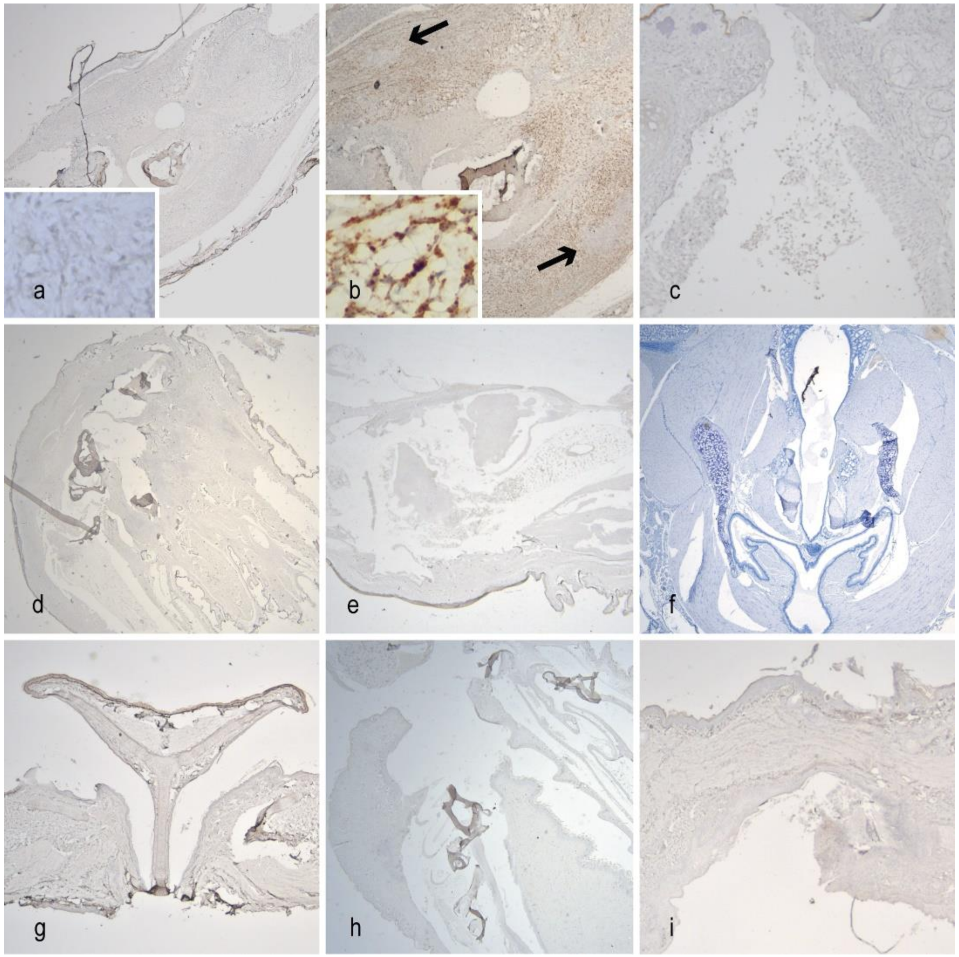

Supplemental Figure S1. Big brown bat eptesipox virus *in situ* hybridization controls, select micrographs. (a) Left 3rd metacarpophalangeal joint from the index case 10, negative control *DapB* probe. Inset with higher magnification demonstrating no background probe binding. (b) Left 3rd metacarpophalangeal joint from the index case 10, positive technical control probe *Eptesicus fuscus PPIB* binding. Arrows indicate regions with low intensity probe binding and these regions correspond to where the poxvirus probes bound in the serial section. Inset with higher magnification demonstrating the morphology of these spindle cells. (c) Pharynx from case with epithelial intracytoplasmic inclusion bodies, case 3, negative control showing no *DapB* probe binding. (d-i). Tissues from control bats treated with poxvirus probes. (d) Carpus, case 9. (e) Elbow, case 4. (f) Oropharynx, case 6. (g) Nose, case 12. (h and i) Ulcers of the oral mucosa of cases 13 and 12, respectively. No eptesipox virus probe binding was detected in any of the controls.

Supplemental Table S1. Big brown bat cases subjected to *in situ* hybridization for eptesipox virus from Saskatchewan, Canada.

ID	Age	Sex	Weight	City	Date Found	Death Date	Necropsy date	History	Tissues ISH	Tissues HE	Diagnosis
1	Adult	Male	12 g	Saskatoon	2019-04-21	2019-04-22	2019-04-22	Found on sidewalk and appeared weak. Submitted live.	Nose, ear, lung, kidney, lymph node, adrenal gland, and brain.	Lung, liver, kidney, spleen, brain, spinal cord, thyroid gland, adrenal gland, oral cavity, esophagus, stomach, small intestine, nose, trachea, patagium, ear, and heart.	Pulmonary hemorrhage and hepatic necrosis - blunt trauma
2	Adult	Female	13 g	Vonda	2019-04-19	2019-04-19	2019-04-23	Found injured on street. Large wing tear and ulcer of palate noted on exam. Euthanized IV with Euthanyl, froze.	Stomach, brain, ovary, uterus, urinary bladder, trachea, esophagus, and small intestine.	Lung, liver, kidney, spleen, brain, spinal cord, ovary, stomach, intestine, colon, nose, trachea, patagium, ear, heart, and humerus.	1. Trauma<br>2. Erosive tracheitis, mild with intranuclear epithelial inclusion bodies
3[a]	Adult	Male	N/A	Saskatoon	2019-02-28	2019-05-13	2019-05-13	In rehab since Feb 28, 2019. Nose depigmented, spots on wings, white exudate in nostrils. Unable to fly. Submitted live.	Head with pharynx, and spinal cord.	Lung, liver, kidney, spleen, brain, spinal cord, adrenal gland, oral cavity, stomach, small intestine, nose, patagium, and heart.	Ulcerative dermatitis and stomatitis with intracytoplasmic inclusion bodies, Eptesipox virus
4	Adult	Female	15 g	Saskatoon	2019-05-15	2019-05-16	2019-05-16	Found in building stair well, right wing compromised. Submitted live.	Elbow and spinal cord.	Lung, liver, kidney, spleen, brain, spinal cord, adrenal gland, lymph node, stomach, intestine, ovary, nose, trachea, patagium, ear, heart, and right elbow.	Chronic suppurative arthritis of right elbow
5	Adult	Male	12 g	Martensville	2019-12-20	2020-03-13	2020-03-13	In rehab since December 20, found under a coke machine. Swollen lips/wrists, started Metacam and liquid diet, March 1, started Baytril, Mar 12, very lethargic. Submitted live.	Nose, stomach, pancreas, intestine, heart, and accessory sex gland.	Lung, liver, kidney, spleen, brain, spinal cord, stomach, small intestine, pancreas, nose, patagium, ear, and heart.	Dermatitis of nose and wing
6	Adult	Female	15 g	Saskatoon	2020-04-25	2020-04-27	2020-04-27	Found on the ground, paralysis of left leg. Submitted live.	Head with pharynx, patagium, and spinal cord.	Lung, liver, kidney, spleen, brain, spinal cord, adrenal gland, lymph node, uterus, urinary bladder, oral cavity, stomach, small intestine, colon, nose, trachea, patagium, ear, heart, and humerus.	1. Trauma<br>2. Neutrophilic cellulitis of chin
7	Adult	Female	14 g	Saskatoon	2020-04-28	2020-04-29	2020-04-29	Found in room. Submitted live.	Liver, small intestine, lymph node, kidney, urinary bladder, heart, lung, and spleen.	Lung, liver, kidney, spleen, brain, spinal cord, adrenal gland, lymph node, uterus, urinary bladder, oral cavity, stomach, gall bladder, small intestine, colon, patagium, heart, and humerus.	Hepatic necrosis - trauma
8	Adult	Female	15 g	Saskatoon	2020-04-09	2020-05-04	2020-05-04	In rehab since April 9. Right wing bones swollen given Metacam and Veraflex for 8 days. Swelling resolved, hind feet unable to grip. Submitted live.	Liver, stomach, small intestine, heart, trachea, and pancreas.	Lung, liver, kidney, spleen, brain, ovary, intestine, stomach, small intestine, pancreas, colon, urinary bladder, uterus, nose, trachea, patagium, ear, heart and humerus.	1. Osteopenia<br>2. Trachea cytomegaly and dysplasia with intranuclear inclusion bodies
9	Adult	Male	13 g	Saskatoon	N/A	2020-07-04	2020-07-06	Presented with fractured left humerus, Finder had provided animal with Metacam. Euthanized with Euthanyl IV. Submitted fresh.	Carpus, nose, stomach, wing, liver, and small intestine	Lung, liver, kidney, spleen, brain, spinal cord, thymus, testis, urinary bladder, esophagus, stomach, small intestine, colon, nose, trachea, patagium, ear, heart, humerus, and right carpus.	Trauma
10[a]	Adult	Male	12 g	Saskatoon	2020-06-28	2020-07-08	2020-07-08	In rehab since June 28. Found grounded, abscess left lower canine, phalangeal joints swollen, underweight. Feet no longer gripping. Submitted live.	Mandible, head with oropharynx, spinal cord, elbow, interphalangeal joint, carpus, and foot.	Lung, liver, kidney, spleen, brain, spinal cord, adrenal gland, oral cavity, stomach, gall bladder, small intestine, pancreas, colon, urinary bladder, nose, trachea, patagium, ear, and heart.	1. Fibrinosuppurative and proliferative polyarthritis<br>2. Ulcerative stomatitis with intracytoplasmic inclusion bodies, Eptesipox virus
11	Adult	Female	22 g	Saskatoon	2020-07-29	2020-07-29	2020-07-30	Multiple injuries to wings and dorsum. Suspect predator attack. Euthanized via IV Euthanyl. Submitted fresh.	Liver, patagium, ear, small intestine, vertebral column.	Lung, liver, kidney, spleen, brain, spinal cord, ovary, urinary bladder, small intestine, pancreas, nose, patagium, ear, heart, and femur.	Trauma
12	Juvenile	Male	12.7 g	Dalmeny	2020-09-16	2020-09-29	2020-09-29	In rehab since September 16. Bitey and noisy. Inappetant. Submitted live.	Nose, ear, patagium, pancreas, kidney, lymph node, and small intestine.	Lung, liver, kidney, spleen, brain, spinal cord, stomach, small intestine, pancreas, testes, nose, patagium, tail, ear, heart, and humerus.	Rabies encephalitis
13	Adult	Male	12.9 g	Saskatoon	2020-11-23	2020-12-09	2020-12-09	In rehab since November 23. Found in snow, hard to feed with frost bite on wingtips. Deteriorating. Submitted live.	head with oral cavity, patagium, vertebral column, testis, spinal cord, and esophagus.	Lung, liver, kidney, spleen, brain, spinal cord, oral cavity, stomach, small intestine, nose, trachea, patagium, ear, and heart.	1. Nose, lower jaw multiple ulcers with bacteria and bacterial pulmonary emboli<br>2. Hepatic necrosis
14	N/A	Female	14.9 g	Saskatoon	2020-11-10	2021-01-14	2021-01-15	In rehab since November 10. Unspecified wounds. Submitted live.	Stomach, small intestine, heart, kidney, lymph node, adrenal gland, lung, esophagus, spleen, and pancreas.	Lung, liver, kidney, spleen, brain, adrenal gland, lymph node, salivary gland, esophagus, stomach, small intestine, pancreas, nose, trachea, patagium, ear, and heart.	Trauma
15	N/A	Female	14.7 g	Saskatoon	2020-11-10	2021-01-14	2021-01-15	In rehab since November 10. Unspecified wounds. Submitted live.	Patagium with bone, ear, skeletal muscle, salivary gland, trachea, and esophagus.	Lung, liver, kidney, spleen, brain, spinal cord, lymph node, salivary gland, esophagus, stomach, intestine, urinary bladder, nose, trachea, patagium, ear, heart.	Trauma
16	Adult	Male	14.6 g	Saskatoon	2021-01-07	2021-01-18	2021-01-19	In rehab since January 7. Found in building. Drools/foams at mouth when fed. Submitted live.	Liver, lung, heart, spleen, and esophagus.	Lung, liver, kidney, spleen, brain, spinal cord, adrenal glands, oral cavity, intestine, pancreas, patagium, ear, heart, and left carpus.	Ulcerative cellulitis and dermatitis with intralesional bacteria, pulmonary bacteria emboli, and septic arthirits

[a]PCR positive for Eptesipox viral type A inclusion protein and *in situ* hybridization labeling for Eptesipox virus. [b]Index case from which Eptesipox virus/Saskatoon/01/2020 was isolated. Abbreviations: N/A, not available; ISH, in situ hybridization; HE, hematoxylin and eosin
